# Effect of Pre-Sowing Seed Stimulation on Maize Seedling Vigour

**DOI:** 10.3390/ijms252212480

**Published:** 2024-11-20

**Authors:** Paulina Pipiak, Katarzyna Sieczyńska, Dorota Gendaszewska, Monika Skwarek-Fadecka

**Affiliations:** 1 Łukasiewicz Research Network—Lodz Institute of Technology, 73 Zgierska Str., 91-463 Łódź, Poland; katarzyna.sieczynska@lit.lukasiewicz.gov.pl (K.S.); dorota.gendaszewska@lit.lukasiewicz.gov.pl (D.G.); 2 Department of Plant Ecophysiology, Faculty of Biology and Environmental Protection, University of Lodz, Banacha 12/16, 90-237 Łódź, Poland; monika.skwarek.fadecka@biol.uni.lodz.pl

**Keywords:** maize, waste protein hydrolysates, elemental components, photosynthetic pigments

## Abstract

The aim of this study was to investigate the effects of treating maize (*Zea mays* L.) seeds with fish collagen hydrolysate (FC) and keratin (KE) derived from animal waste by-products of leather and meat production, as well as poly(hexamethylene biguanide) hydrochloride (P) and bentonite (B). This research is in line with the search for new, environmentally friendly methods to increase yields of industrial crops in a way that is compatible with sustainable development. The effect of the binders used was investigated by analysing the grown maize seedlings by determining changes in parameters of chlorophyll fluorescence, photosynthetic pigments, elemental composition and FTIR analysis on maize shoots. The results indicated a slightly higher fresh weight (FW) of shoots in plants treated with fish collagen, PHMB and bentonite (FC+P+B) and FW of roots in plants treated with keratin, PHMB and bentonite (KE+P+B). Unexpectedly, the FW and dry weight (DW) of both roots and shoots of all bentonite-treated plants were significantly higher than the corresponding non-bentonite-treated groups. In addition, changes in chlorophyll-a fluorescence were observed for the keratin, PHMB and bentonite variants. This study showed that the proposed materials could be promising seed pelleting agents to improve seed growth and yield.

## 1. Introduction

Maize (*Zea mays* L.) is the third most widely cultivated crop in the world, and in terms of yield, it is the most productive cereal crop after wheat and rice [1,2]. The great economic importance of maize cultivation is due to its potential for multiple uses, including feed, food and energy [3]. For economically important crops, it is particularly important to implement measures to increase their fertility. This can be achieved through the parallel application of two strategies: the improvement of crop varieties and the development of more efficient and environmentally friendly farming systems [4]. However, with the depletion of natural resources and the degradation of ecosystems, the implementation of sustainable farming practices, including the use of biostimulants, is becoming increasingly important in modern agricultural practices [5]. In addition, closed-loop solutions are being implemented in modern agriculture with the aim of reusing products derived from waste [6]. In this context, the recovery of valuable biopolymers, such as keratin, collagen and elastin, from waste materials generated by the meat industry, the tanning industry and fish waste, is of particular importance [7]. The disposal of this type of animal waste in large quantities represents a significant environmental problem on a global scale. It is estimated that the leather industry generates approximately 6 million tonnes of solid waste per year [8]. In contrast, available data indicate that 23% of production in the meat sector is lost and wasted, of which 20% is waste generated at the production stage [9]. Efficient use of this waste can not only reduce its negative impact on the environment, but also provide valuable raw materials for the production of biopolymers [10,11].

The basic structural units of all living organisms are proteins, of which fibrillar proteins, including collagen, keratin, elastin and fibrin, are of particular importance. Collagen, the building protein of mammals, accounts for more than 30% of the total protein composition of the body. It is an essential component of the skin, teeth, bones, tendons, as well as cartilage and the cornea of the eye [12,13]. In contrast, in skin products such as wool and feathers, keratin can account for up to 90% of the total weight of the material [14]. The high content of these biopolymers in materials that are by-products and biomass wastes from the agri-food industry makes their extraction possible and thus encourages the development of methods for their processing. As a result of these developments, collagen and keratin are becoming increasingly important not only in the field of medicine but also in other sectors such as agriculture [15]. Collagen and keratin are a valuable source of essential amino acids for plant growth. Amino acids such as threonine, proline, glycine, aspartic acid and glutamic acid play a key role in plant metabolism, facilitating the synthesis of proteins, hormones and enzymes. These processes directly influence the healthy development and vigour of plants [16]. The use of collagen or keratin hydrolysates as biostimulants in the cultivation of selected crops can increase the bioavailability of these nutrients, thereby contributing to increased yields and superior crop quality.

In the context of economically important crops, it would be beneficial to investigate the effect of pre-sowing seed stimulation on plant development and yield. Seed quality and treatment are among the most important factors influencing yield [17,18]. This method has the potential to significantly influence the nutrient content of maize shoots [19]. It also offers an alternative to conventional fertiliser (nutrient) application methods, thereby increasing maize yields even under drought conditions and increasing seed emergence potential [20,21].

Despite the documented benefits of protein hydrolysates and amino acids on plant growth when applied as foliar preparations, their use in seed pelleting remains a subject of ongoing investigation. This is particularly relevant in the context of the search for pro-ecological methods to increase the yield of economically important crops in a manner consistent with sustainable development. The aim of this study was to investigate the effects of encapsulating maize seeds with selected biopolymers (fish collagen, keratin), the poly(hexamethylene biguanide) biocide PHMB and bentonite on the grown and development of maize seedlings. This work is a continuation of previous studies on the effects of seed pelleting with waste biopolymers (bovine collagen and fish collagen) and the PHMB biocide [22,23]. The promising results obtained with seed stimulation with both fish collagen and the biocide provided the impetus to continue work on this topic and encouraged the testing of the biostimulatory effect of keratin hydrolysates, PHMB and bentonite.

This research is part of a wider strategy to develop modern seed coating methods that minimise environmental impact. This includes reducing the use of agrochemicals and lowering process costs. In addition, the use of products derived from waste biomass is in line with the principles of a circular economy.

## 2. Results

### 2.1. Effect of Combined Application of Fish Collagen, Keratin, PHMB and Bentonite on FW and DW Contents of Maize Seedlings

**Figure 1:**
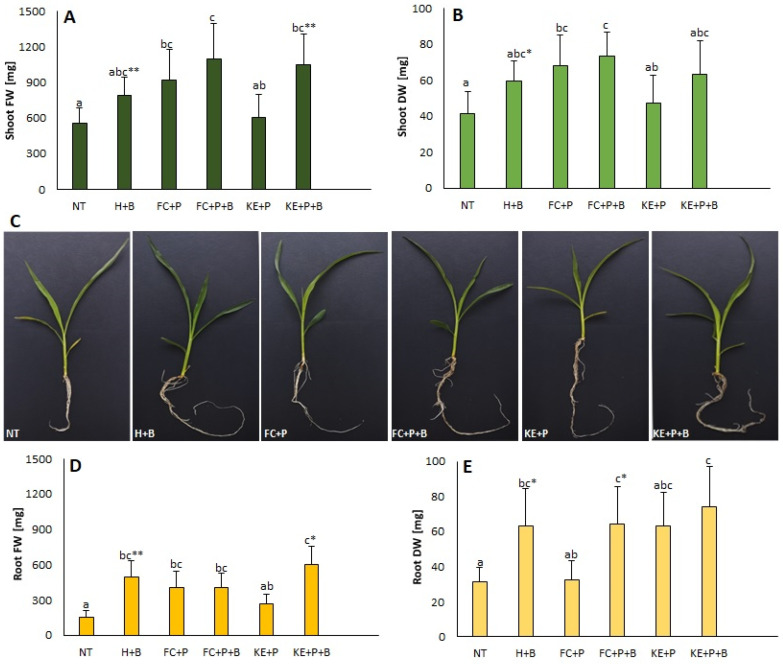
Fresh weight (FW; (**A**)) and dry weight (DW; (**B**)) of shoots and fresh weight (FW; (**D**)) and dry weight (DW; (**E**)) of roots of 21-day-old maize plants (**C**) subjected to different seed treatments. Values followed by different letters within a given binding agent treatment (NT, FC+P or KE+P) are significantly different (*p* < 0.05; ANOVA followed by Tukey’s post hoc test; *n* = 8); values within a given bentonite treatment (H+B, FC+P+B or KE+P+B) are significantly different (* for *p* < 0.05, ** for *p* < 0.01; Student’s *t*-test; *n* = 8). NT—non-treated seeds, FC—fish collagen-treated seeds, KE—keratin-treated seeds, P-PHMB—poly(hexamethylene biguanide) hydrochloride-treated seeds, H—water-treated seeds, B—bentonite-treated seeds.

The significant changes in fresh weight (FW) and dry weight (DW) were observed in the treated shoots and roots compared to the respective controls (NT) (Figure 1). In addition, slightly higher FWs of shoots in FC+P+B-treated plants (about 49% compared to the control) (Figure 1A) and FWs of roots in KE+P+B-treated plants (about 74% compared to the control) (Figure 1D) were recorded. Unexpectedly, the FW and DW (Figure 1B,E) of both roots and shoots of all bentonite-treated plants (H+B, FC+P+B, KE+P+B) were significantly higher compared to the respective non-bentonite-treated groups (NT, FC+P, KE+P). In addition to the binder, the FW and DW of both roots and shoots were also dependent on bentonite. Figure 1C shows images of representative seedlings subjected to different seed coating treatments.

### 2.2. Changes in Elemental Composition

The analysis showed significant effects of the use of binding agents and bentonite on the elemental composition of maize seeds, roots and shoots (Table 1). The results between the bentonite and non-bentonite variants also showed significant differences in elemental content.

Significantly more Ca, Cu, Fe and Na were found in the seed composition of plants treated with binding agents and bentonite than in non-treated plants. It is interesting to note that only the seeds that have been treated with additional bentonite contain Cr. Furthermore, the FC+P, FC+P+B, KE+P and KE+P+B seeds contain less K than NT and H+B seeds. On the other hand, the FC+P, FC+P+B, KE+P and KE+P+B seeds have a lower K content than the NT and H+B seeds. There was a significant increase in Mn content in the H+B and FC+P+B seeds.

Regarding root composition, the most significant changes were observed in the Ca, Cu, Mn, Fe, P and Zn contents of plants treated with binding agents and bentonite compared to non-treated plants. The Na content did not change significantly after treatment with binding agents. Only the FC+P treatment was higher than the control. The FC+P+B and KE+P+B roots contain less K than the NT roots. Cr was present in the roots of all variants analysed. Particularly high levels of Cr were found in FC+P+B, KE+P and KE+P+B.

The shoots also showed some significant changes. FC+P+B and KE+P contained significantly more Cu than the other samples. The results for all the variants tested, with the exception of KE+P+B, also show a high Zn content. It is interesting to note that the results show a decrease in the content of some of the elements after the application of the binding agents or bentonite. After the application of fish collagen, there is a significant reduction in the content of Ca, Cu, Cr, Fe, K, Na, P in the shoots. The content of elements such as K, Na and P in the shoots is reduced by the use of keratin as a binder. The results indicate that K, Mg, P and Zn are the most abundant elements in the seedlings of the tested plant.

### 2.3. Changes in Chlorophyll Fluorescence Parameters

**Figure 2:**
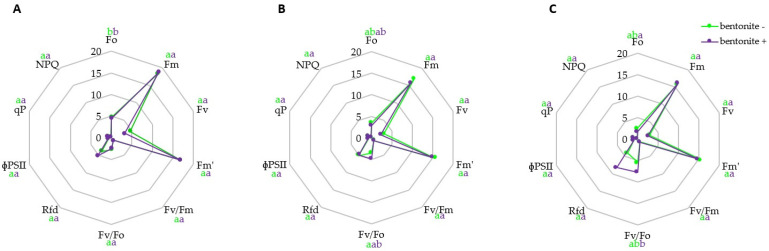
Changes in chlorophyll fluorescence parameters in 21−day−old maize plants exposed to different seed coating treatments. Values followed by different letters within the given binding agent treatment (NT; (**A**), FC+P; (**B**) or KE+P; (**C**)) are significantly different (*p* < 0.05; ANOVA followed by Tukey’s post hoc test; *n* = 5). The colour of the letter-based statistical indicators refers to each experimental variant as indicated in the legend. Abbreviations: Fo−basic fluorescence, Fm−maximal fluorescence, Fv−maximal variable fluorescence, Fm′−maximal fluorescence for the light-adapted state, Fv/Fm−maximum photochemical quantum yield of PSII in the dark-adapted state, Fv/Fo−efficiency of the water-splitting complex on the donor side of PSII, Rfd−vitality index, ФPSII—quantum efficiency of PSII, qP−photochemical fluorescence quenching, NPQ−non-photochemical fluorescence quenching.

Small changes in chlorophyll-a fluorescence were observed (Figure 2). The Fv/Fm ratio, which estimates the maximum quantum efficiency of PSII photochemistry, was higher in KE+P+B plants compared to the control, possibly indicating an increase in photosynthetic activity. In addition, analysis of PAM chlorophyll fluorescence signals revealed significant changes in the balance between photochemical and non-photochemical processes in PSII (Fv/Fo) in the KE+P+B variant. However, there were no significant changes in the maximum fluorescence levels, Fm and Fm′, in dark-acclimated and light-exposed samples in all plants. The data also showed that PAM parameters (Fv, Rfd, ФPSII, qP, NPQ) in the binder- and bentonite-treated plants remained similar to those in the non-treated plants.

### 2.4. Changes in Photosynthetic Pigments Content

**Figure 3:**
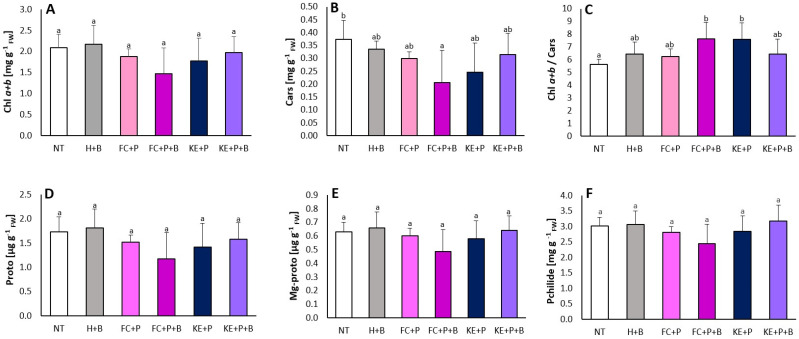
Content of chlorophyll *a + b* (Chl *a + b*) (**A**), carotenoids (Cars) (**B**), ratio of chlorophyll *a + b* to carotenoids (Chl *a + b*/Cars) (**C**), porphyrins: protoporphyrin (Proto) (**D**), Mg−protoporphyrin (Mg−proto) (**E**) and protochlorophyllide (Pchlide) (**F**) in leaf discs of 21−day−old maize plants. Values followed by different letters within a given binding agent treatment (NT, FC+P or KE+P) are significantly different (*p* < 0.05; ANOVA followed by Duncan’s post hoc test; *n* = 4). NT−non-treated seeds, FC−fish collagen-treated seeds, KE−keratin-treated seeds, P−PHMB-poly(hexamethylene biguanide) hydrochloride-treated seeds, H−water-treated seeds, B−bentonite-treated seeds.

The analysis showed some significant changes in the ratio of chlorophyll *a + b* and carotenoids and carotenoid content and no significant changes in chlorophyll *a + b* and porphyrin content (Figure 3). For chlorophyll *a + b*, carotenoids and protoporphyrin, the highest changes were observed in the H+B treated plants. The lowest significant changes were observed for carotenoids in FC+P+B. Higher levels of Mg-protoporphyrin and protochlorophyllide were observed in H+B- and KE+P+B-treated plants compared to the control. The ratio of Chl *a + b* and Cars was the highest in the FC+P+B and KE+P treatments. The ratio of Chl *a + b* and Cars was almost the same in the other variants studied. The highest significant values were observed in FC+P+B and KE+P.

### 2.5. FTIR Analysis on Maize Shoots

This work also included an analysis of dried maize seedling shoots by Fourier-transform infrared spectroscopy in attenuated total reflectance mode (ATR-FTIR). In this study, dried maize seedling shoots (laboratory dryer, 70 °C, 24 h) were analysed by ATR-FTIR. It is important to note that the quality of the ATR measurement is highly dependent on the direct contact between the sample and the crystal surface [24,25]. For this reason, a uniform gauge force amount of 100–110 was applied to the plant samples tested.

**Figure 4:**
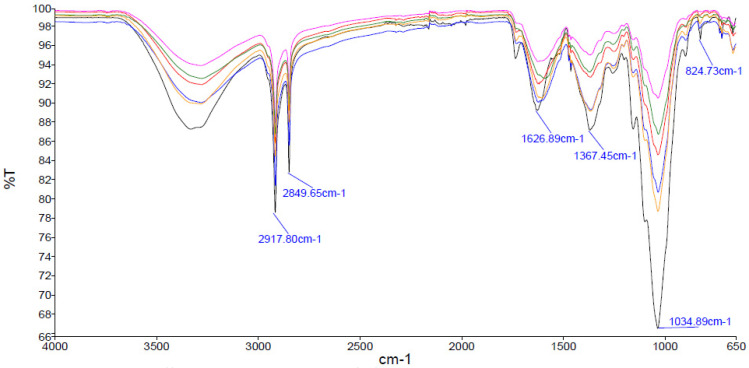
FTIR spectra of 21−day−old maize leaves of the tested variants and the control sample: NT (black line), H+B (red line), FC+B (blue line), FC+P+B (pink line), KE+P (green line), KE+P+B (yellow line).

The recorded ATR-FTIR spectra of the dried maize seedling samples following modification were compared with those of the control sample (NT) to identify any differences in maximum absorbance values and peak positions (Figure 4). The presence of bands characteristic of cellulose, hemicellulose and lignin was observed in all the samples recorded. A broad band at 3287 cm^−1^ was identified as O-H stretching, which occurs in cellulose, hemicellulose and lignin [26,27,28]. Bands at 2849–2917 cm^−1^ were attributed to C-H stretching in methyl and methylene groups in cellulose, hemicellulose and lignin [28,29,30]. The band at 1732 cm^−1^ corresponds to C=O stretching in hemicellulose and lignin [28,30]. It is likely that the intense band at 1626 cm^−1^ is indicative of C-H-C vibrations in water absorbed on cellulose [31]. Furthermore, the presence of cellulose is confirmed by the presence of bands at 1367 cm^−1^ C-C stretching, 1155 cm^−1^ C-O unsymmetrical stretching in cellulose, among others [32]. Nevertheless, the presence of hemicellulose is confirmed by the observation of vibrations at 1462 cm^−1^ CH_2_ symmetric bending, 1367 cm^−1^ C-H bending, CH_3_ symmetric bending and 1099 cm^−1^ asymmetric in-phase ring vibration [27,30]. Lignin is characterised by a number of distinct spectral features, including bands of C=O stretching at 1245 cm^−1^, C-C, C-O C=O vibration at 1201 cm^−1^ and C-H phase deformation vibration at 1034 cm^−1^ [28].

The presence of proteins is confirmed by the observation of bands at 1750–1200 cm^−1^, which are attributed to amides and amine bands [33]. The bands at 1700–1600, which correspond to the C=O group in amide I, the bands at 1600–1500, which correspond to vibrations of the C-N group in amine I and amide II, and the band at 1400–1200, which corresponds to the C-N-H group in amine II and amide III, respectively [34].

## 3. Discussion

In the study, plants treated with fish collagen and additives (FC+P+B) showed a slight increase in shoot fresh weight (FW), while those treated with keratin extract and additives (KE+P+B) exhibited a slight increase in root fresh weight. This may be due to a suitable amino acid profile of the biopolymers tested and the inclusion of bentonite in the seed-coating formulation. The fish collagen selected for the study contains predominantly glycine (2768 mg kg^−1^), proline (3606 mg kg^−1^) and hydroxyproline (1773 mg kg^−1^) in its composition. Analysis of the amino acid profile of the keratin hydrolysate showed the highest content of glutamic acid (Glu) at 888 mg kg^−1^. Compared to the fish hydrolysate, the keratin hydrolysate contained higher amounts of valine, leucine, isoleucine, threonine, serine, methionine and phenylalanine. The role and importance of the aforementioned amino acids in plant growth and development has been confirmed in the literature [35,36], which was also proven in the present study. It is worth mentioning that previous works showed that treatment of pea seeds with bovine collagen hydrolysate, fish collagen hydrolysate and PHMB had a major effect on plant growth, while at the same time dolomite had a negative effect through differences in the morphological characteristics of the stipules [23]. In contrast, analysis of the elemental composition of pea roots and shoots showed a positive effect of the applied fish collagen hydrolysate and PHMB in pea cultivation and a negative effect of bovine collagen hydrolysate and dolomite [23].

Therefore, the slightly higher shoot FW values obtained in this study in plants treated with fish collagen and additives (FC+P+B) may be due to the biostimulatory properties of FC. The stimulating properties of FC on plant germination and growth have only been investigated to a limited extent [37]. A study by Bhagwat et al. confirmed the efficacy of a water-soluble fish collagen extract in promoting the seed germination of *Vigna radiata* [38]. Other work has also shown that fish hydrolysates improve nutrient use by plants and induce morphological changes in root architecture [39,40]. At the same time, the results obtained from a slightly higher root FW value in plants treated with keratin extract and additives (KE+P+B) are consistent with the few data available in the literature on the effect of keratin application on plant growth [41,42,43]. The work of Bhavsar et al. [44] showed a positive effect of keratin hydrolysate on an increase in the number of germinating seeds (11.9%) and an increase in root length (58.2%) [44]. Another study demonstrated the biostimulatory properties of using keratin hydrolysate on maize plants [45]. However, in this case, the authors used both seed coating with enzymatic keratin dispersions and foliar application of the plants with keratin hydrolysate [45]. The greenhouse trials showed an increase of 8.4–19% in plant length and chlorophyll concentration compared to the control samples [45]. In relation to the studies conducted in this thesis, this may suggest that the magnitude of the response to hydrolysate application is dependent on its concentration [42].

In the tests carried out, the use of bentonite as a berm material proved to be more beneficial to the growth of the plants tested. Bentonite is a natural, non-toxic soil amendment that retains water and nutrients during rainfall and releases them during dry periods [46]. According to the literature, the use of such superabsorbent polymers is an effective method of optimising water use and improving crop yield [47]. In the study by Mi et al. [46], field experiments were conducted on millet to evaluate the effects of bentonite amendments on soil biochemical properties. The increase in soil microbial activity and nutrient availability and cycling was attributed to higher soil water content due to the presence of bentonite. This resulted in improved plant growth and the incorporation of organic matter into the soil, providing more substrate for microorganisms. Therefore, the use of bentonite as a soil amendment can promote sustainable agricultural production.

Analysis of the elemental composition of seeds treated with fish collagen, keratin, PHMB and bentonite showed changes in the amount of elements following the seed-coating process. The germination stage of seeds and the early stages of seedling development are important for the yield of industrial crops. In this context, the amount of micro- and macronutrients present in the seed is of great importance. The analysis revealed that the seeds from the H+B, FC+P+B and KE+P+B treatments had a calcium ion content (0.24–0.25 mg/kg) more than double that of the control sample (0.10 mg/kg) (Table 1). Additionally, elevated levels of sodium iodine were found in the identical treatments (0.37–057 mg/kg, Table 1). The observed changes in the contents of these elements are probably due to the use of bentonite, a mineral substance characterised by significant amounts of calcium and sodium. As shown in the literature, the incorporation of exogenous calcium ions, achieved by seed treatment, has been shown to alleviate the adverse effects of salt stress [48]. The presence of calcium ions has been shown to improve seed germination by maintaining ion homeostasis in the presence of salt stress [49,50,51,52]. In contrast, the presence of sodium at low concentrations may be particularly important for plants under conditions of K+ deficiency [53,54,55]. On the other hand, in the seed samples tested, the highest contents of copper and iron were observed in the seeds coated with the KE+P+B variant, 6.5 mg/kg and 0.14 g/kg, respectively.

In the case of roots, the elemental composition analysis showed differences in the Ca, Cu, Mn and Zn contents depending on the experimental variant analysed, with the highest contents of the above-mentioned elements observed for the KE+P and KE+P+B variants (Table 1). The physiological functions of these elements in plants clearly demonstrate their involvement in many physiological processes of fundamental importance for plants. The role of copper in plant metabolism is closely linked to its contribution to the enzyme systems involved in oxidoreductive processes [56]. According to the literature, Cu nanoparticles can enter the plant cell through roots and leaves, and their bioaccumulation increases with increasing ion concentration [57,58]. Zinc, in turn, is an essential element that activates a number of plant enzymes, including those involved in protein synthesis, carbohydrate metabolism, regulating auxin synthesis and influencing pollen formation [59]. It is also an essential element in maintaining the integrity of cell membranes and influences the ion transport system [60]. Furthermore, research indicates a correlation between zinc (Zn) and the evolution of plant defence mechanisms and the mitigation of disease severity [61]. Manganese is another element that affects plant metabolism. It is a component of many enzymes and influences the efficiency of enzyme-catalysed reactions, such as redox reactions, phosphorylation, hydrolysis or decarboxylation [62].

In the last few years, the measurement of chlorophyll fluorescence parameters has emerged as a rapid, non-invasive technique to accurately assess the state of the photosynthetic apparatus, in particular photosystem II (PSII) [63,64]. The Fv/Fm ratio indicates the maximum photochemical quantum yield of PSII. Values between 0.75 and 0.85 are considered normal in non-stressed plants [65]. The Fv/Fo ratio is more sensitive in general because it expresses the efficiency of the water-splitting complex on the donor side of PSII, which is the most sensitive component in the photosynthetic electron transport chain [66]. It provides similar fundamental data but has higher values and a wider dynamic range than Fv/Fm [67]. This is in agreement with the present results, especially in leaves treated with keratin, PHMB and bentonite (KE+P+B), which show that the changes in the Fv/Fo ratio are faster and greater than those in the Fv/Fm ratio, which could be related to the increase in photosynthetic activity. Moreover, photosynthetic processes, including PSII photochemistry, can be affected by nutrient deficiencies, which directly affect the photosynthetic apparatus through the synthesis and function of essential photosynthetic components [68]. Nitrogen, sulphur and iron deficiencies can directly affect the synthesis of protein complexes involved in photosynthetic reactions [69]. Potassium is crucial for stomatal function as it helps to regulate turgor pressure [70] Sharma et al. [71] highlighted the important role of zinc in regulating stomatal aperture, suggesting that zinc may contribute to maintaining high levels of potassium in guard cells. The chlorophyll fluorescence parameters (Fv/Fm, ΦPSII, NPQ and qP) were observed to be highly variable, with higher values in the midrib region of the strawberry at day 42 of iron-deficient conditions, as reported by Osório et al. [72]. In the present data, we did not observe any changes in the PAM parameters (Fv, Rfd, ФPSII, qP, NPQ) in plants treated with binder and bentonite compared to untreated plants, which may indicate the proper photosynthetic state of the plant.

In addition, ATR-FTIR analysis was performed to verify differences in the chemical composition of the cell walls of the plant material tested. In the case of plant samples, FTIR allows changes in the proportions of the major organic compounds present in the plant material to be analysed, both between different plant species and within the same species [73]. The main constituents of maize leaves are cellulose and hemicellulose, which together account for about 50% of the total biomass on a dry weight basis. According to the literature, the cellulose content ranges from 32 to 40%, while the hemicellulose content ranges from 18 to 25% [74,75,76]. In addition, maize leaves contain a significant amount of lignin, ranging from 11 to 17% of the dry biomass [74,75,76]. In addition to the carbohydrates mentioned above, leaves also contain proteins, lipids, chlorophyll and mineral nutrients. The chemical composition of leaves is influenced by a number of factors, including the stage of development of the plant and the cultivation methods used. In the recorded spectra for samples FC+PHMB and KE+PHMB + B, an increase in band intensity was observed in the 1450–1300 cm^−1^ region. This is consistent with the data described previously, which indicated the presence of vibrations of the C-C groups in cellulose and the C-N-H groups in amine II and amide III in this range. The observed relationships may be the result of changes in the amide and amino acid content of plant samples whose seeds were treated with protein solutions (FC—fish collagen and KE—keratin, respectively).

## 4. Materials and Methods

### 4.1. Plant Material

The sweetcorn seeds used in all studies were of the Gucio F1 variety obtained from the Horticultural Seed and Nursery Company in Ozarow Mazowiecki (Ozarow Mazowiecki, Poland, PNOS Ltd.). Gucio F1 sweetcorn is an early hybrid variety that is known for its exceptionally sweet taste. The growing period of this sweetcorn is about 110–120 days. It is grown for use in the food industry. Importantly, it retains its sweet taste and intense colour even after processing.

### 4.2. Coatings

#### 4.2.1. Hydrolysed Fish Collagen (FC) and Keratin (KE)

Fish collagen was obtained from the skin of *Hypophthalmichthys* sp. (Cyprinidae) (INVENTIA Polish Technologies, Żuławka, Poland). Keratin hydrolysate was obtained from sheep wool (PROTEINA, Natural Protein Factory, Łódź, Poland). The chemical properties of the biopolymers used in the study were characterised. The properties of the fish collagen used were previously described in the work of [23]. However, in order to systemise and compare the data obtained for keratin, the characteristics of both biopolymers are given in Table 2. The chemical properties of fish collagen and keratin were studied using quantitative methods, including weighing and potentiometric methods, titration method (Kjeldahl method) and GC-MS. The amino acid content was determined by gas chromatography (GC) according to accredited analytical method PB 5.4, ed. 4 (30 June 2013). The content of each metal was determined using dedicated techniques: vapour generation atomic absorption spectrometry (VGAAS) for the determination of As and Hg and flame atomic absorption spectrometry (FAAS) for the determination of Cr, Zn, Pb, Cd and Cu. The concentrations of individual metals were determined using the following techniques:

1. VGAAS in this:
  (a) Hydride Generation Atomic Absorption Spectrometry (HGAAS)—atomic absorption spectrometry with hydride generation, used to detect metal content, e.g., As. In this method, a so-called hydride generator is used for determinations to produce volatile metal–hydrogen compounds.
  (b) Cold Vapour Atomic Absorption Spectrometry (CVAAS)—cold vapor atomic absorption spectrometry for Hg metal. A technique used only for Hg determinations. A hydride generator is also used for research, producing free mercury atoms.
2. FAAS—This is a technique using a flame atomizer dedicated to metals, in this case Cr, Zn, Pb, Cd, Cu, which transforms the liquid sample into an aerosol introduced into the flame. The measurements were carried out with hollow cathode lamps (HCLs), using lamps appropriate for the specific element. Flame atomization was used, using background correction (deuterium lamp) for metals: As, Cd, Cr, Cu, Hg, Pb, Zn, using most sensitive analytical lines in the range below 400 nm. The deuterium lamp (D2) emits radiation in a wide range (190–400 nm), which is not absorbed significantly by the sample atoms but is subject to scattering and other background influences. The type of flame, the composition of the gas mixture and the wavelength varied according to the element being determined. A 50 mm long burner was used for the measurements.

**Table 2:** Chemical properties of fish collagen and keratin used for maize seeds coating.

Parameter	Fish Collagen	Keratin
Acidity of 10% solution (pH)	3.46	3.91
Protein content (%)	89.90	57.43
Total nitrogen content (%)	14.30	16.65
Heavy metal content (mg kg^−1^)		
Cr (III)	<0.1	<0.003
Zn	<0.05	<0.01
As	<0.05	<0.02
Pb	<0.10	<0.02
Hg	<0.10	<0.008
Cd	<0.10	<0.005
Cu	<0.02	<0.002
Sn	-	-
Amino acid content (mg kg^−1^)		
Ala	931.3 ± 19.5	319.0 ± 32.0
Gly	2768.3 ± 422.9	303.0 ± 30.0
Val	148.3 ± 6.7	339.0 ± 34.0
Leu	165.7 ±15.0.9	479.0 ± 48.0
Ile	74.0 ± 4.0	204.0 ± 20.4
Thr	228.3 ± 10.4	357.0 ± 36.0
Ser	275.7 ± 5.1	528.0 ± 53.0
Pro	3606.0 ± 129.0	389.0 ± 39.0
Asp	336.7 ± 10.4	415.0 ± 41.0
Met	82.7 ± 1.5	240.0 ± 2.4
Hyp	1773.3 ± 30.5	<0.02
Glu	642.0 ±12.1	888.0 ± 88.0
Phe	140.3 ± 7.5	201.0 ± 20.1
Lys	196.3 ± 1.5	197.0 ± 20.0

#### 4.2.2. Poly(hexamethylenebiguanide) Hydrochloride (PHMB) Biocide

PHMB is an ionic liquid that is commercially available as an aqueous solution (VANTICIL IB, 20% aqueous solution, Lonza, Arch UK Biocides Ltd., Castleford, UK). PHMB is an antiseptic agent with effective bactericidal activity and relatively low cytotoxicity [22,23]. The commercial, aqueous solution of PHMB is a slightly opalescent colourless or pale-yellow liquid with a pH of 4.6 and a density of 1.04 g/cm^3^. The appropriate concentration of this compound has been prepared by diluting the commercial formulation with deionised water.

#### 4.2.3. Bentonite (B)

Commercially available bentonite (BENTONIT SPECJAL, Zakłady Górniczo-Metalowe “ZĘBIEC”, Zębiec, Poland) was used in this study. The main component of bentonite is the clay material montmorillonite. Chemically, bentonite is a hydrated aluminosilicate with strong adsorption properties. In order to characterise the material used, the elemental composition was determined according to the procedure described. Representative samples of the materials analysed were selected. The procedure was as follows: samples weighing approximately 0.2 g of bentonite were weighed in weighing containers, then placed in a Teflon container and 6 cm^3^ of HNO_3_ (65%, wt., Chempur, Piekary Śląskie, Poland) and 1 cm^3^ of HF (40%, wt., Merck, Darmstadt, Germany) were added at 190 °C. The samples were then mineralised using a Magnum II microwave mineraliser (Ertec, Wroclaw, Poland). Digestion was carried out in two steps. In the second digestion step, the HF acid was neutralised with 10 mL of a 4% H_3_BO_3_ solution (cz.d.a., Chempur) at 150 °C. The clear mineralisates were quantitatively transferred to 25 cm^3^ flasks and made up with demineralised water. The chemical composition of the bentonite is given in Table 3. Similarly, reagent blank was prepared.

### 4.3. Experimental Design and Seed-Coating Method

In the study, six variants of seed treatment were developed using binders such as water, fish collagen, keratin and PHMB and also using bentonite (Table 4). The immersion method was used to coat the seeds. Aqueous solutions of binders including 0.4% fish collagen solution, 0.4% keratin solution and 1% PHMB solution, were prepared. The seeds were then immersed in the solutions for 30 min, depending on the experimental variant. An amount of 50 mL of each solution was used to coat 100 maize seeds. In treatment 3–6, where seeds were coated with two substances, the seeds were first coated with either FC or KE, respectively. After draining for about 30 min, the seeds were then immersed in a PHMB solution. For treatment involving the addition of bentonite, the seeds were dried and then placed in a dish containing 5 g of bentonite. The seeds were stirred for approximately 15 min, after which any excess bentonite was removed and the seeds were weighed on an analytical weight balance.

### 4.4. Growth Conditions

Plants were grown in a RoyalRoom growbox (200 × 200 × 100 cm) under controlled laboratory conditions. Growth conditions included 16 h of artificial lighting (Lumatek Attis 200W LED FULL SPECTRUM ATS200W, Lumatek Ltd, St Julian’s, Malta), with a day/night temperature of 21/19 °C and a relative humidity of approximately 50%. The maize was grown in plastic trays of 58 × 40.5 × 7 cm using commercially available horticultural soil (Table 5). The test material consisted of 21-day-old maize seedlings.

### 4.5. Growth Measurements

Growth parameters such as the fresh weight (FW) and dry weight (DW) of leaves of the same size were measured. The FW was measured immediately after harvest. The DW was determined by drying the plant material in paper envelopes at 60 °C for at least 48 h.

### 4.6. Determination of Elemental Composition

The methodology used for the determination of metals in samples of maize shoots and roots has previously been described in the work of Skwarek et al. [23].

The research material consisted of samples of maize shoots and roots placed in a Teflon vessel containing 6 cm^3^ of 65% HNO_3_ (Chempur, Piekary Śląskie, Poland). Samples were mineralised using a Magnum II microwave mineraliser (Ertec, Wroclaw, Poland). The parameters of the mineralisation process included mineralisation at a maximum microwave power in three cycles of a total of 20 min at a maximum temperature of 300 °C and a maximum pressure increasing to 45 bar. The clear mineralisates were then quantitatively transferred to 25 cm^3^ volumetric flasks and made up with demineralised water. Similarly, reagent blank was prepared. Samples were analysed by inductively coupled plasma atomic emission spectrometry using an ICP-OES 5110 spectrometer (Agilent, Santa Clara, CA, USA). Commercially available argon (HenDuKol, Łódź, Poland) was used to generate the plasma. Spectrometer parameters during the analysis: generator power 1400 W, plasma gas (flow rate) 12 dm^3^/min, auxiliary burner cooling gas (flow rate) 1 dm^3^/min, gas in the nebuliser (flow rate) 0.7 dm^3^/min, nebuliser type OneNeb, chamber fog Double Pass Cyclonic Chamber, measurement reading time 3 × 10 s, sample flow rate 1.4 cm^3^/min, limit of correlation coefficient 0.9990.

Calibration of the measuring method was carried out before the measurement using a series of chemical standards (reference materials) with different levels of the content of the tested component. The instrument was calibrated by using standard solutions of studied metals. The content of the tested metals in the samples was read from standard curves prepared from the calibration solution of individual metals (Ca, Cr, Cu, Fe, K, Mg, Mn, Na, Zn in 2–5% HNO_3_; B, P in H_2_O and Sn in 20% HCl Chem-Lab, Zedelgem, Belgium) 1000 mg dm^−3^ by appropriate dilution of the standards HNO_3_, obtaining the HNO_3_ concentration in the standards as in the samples after the mineralization process. The concentration of the calibration solutions for ICP-OES analysis ranged from 0.005 to 200 mgdm^−3^ for the trace elements B, Ca, Cr, Cu, Fe, K, Mg, Mn, Na, P, Sn, Zn. The whole procedure was repeated four times.

#### Bentonite

Samples representative of the materials analysed were selected. The procedure was as follows: samples weighing approximately 0.2 g of bentonite were weighed in weighing vessels, then placed in a Teflon vessel and 6 cm^3^ of HNO_3_ (65%, wt., Chempur) and 1 cm^3^ of HF solution (40%, wt., Merck) were added at 190 °C. The samples were then mineralised using a Magnum II microwave mineraliser (Ertec). Digestion was carried out in two steps. A second digestion step, the HF acid was neutralised with 10 mL of a 4% H_3_BO_3_ solution (cz.d.a., Chempur) at 150 °C. The clear mineralisates were quantitatively transferred to 25 cm^3^ flasks and made up with demineralised water.

### 4.7. Chlorophyll Fluorescence Parameters

Photosynthetic activity was assessed in 21-day-old maize plants grown under the conditions described in Section 4.4. A pulse amplitude modulation (PAM) fluorometer (JUNIOR-PAM, WALZ, Effeltrich, Germany) was used to measure chlorophyll fluorescence, following the manufacturer’s instructions with the WinControl-3 Windows software. Measurements were conducted at room temperature (25 °C) in a dark room illuminated by dim green light to facilitate the process, on the adaxial side of the leaves. Chlorophyll fluorescence parameters were recorded at four locations on five plants of each variant. All measurements began after acclimating the leaves to dark or dim light for 30 min. Basic fluorescence (Fo) was recorded with a low intensity modulated light beam (ML, 200–300 mV) and maximum fluorescence (Fm) was measured after a saturating pulse of white light (SP, 10,000 µmol photons m^−2^ s^−1^ for 0.8 s), closing all reaction centres. The actinic light (AL) was 190 μmol photons m^−2^ s^−2^. The steady-state level (Fs) was estimated after switching to actinic light. The following parameters were calculated: the maximum photochemical quantum yield of PSII (Fv/Fm, where Fv = Fm − Fo) [77]; the efficiency of the water-splitting complex on the donor side of PSII (Fv/Fo); the quantum efficiency of PSII [ΦPSII = (Fm′ − Fs)/Fm′]; the vitality index (Rfd), an indicator of CO_2_ fixation (Fm − Fs)/Fs [67]; photochemical quenching (qP), which represents the fraction of PSII reaction centres in the open state (Fm′ − Fs)/(Fm′ − Fo′) [78]; and non-photochemical quenching [NPQ = (Fm − Fm′)/Fm′], which measures energy dissipation as heat [79].

### 4.8. Photosynthetic Pigment Contents Determination

For chlorophyll, carotenoids and total porphyrin content, 0.1 g of leaves were placed in test tubes and 15 mL of 96% (*v*/*v*) ethanol was added to each tube following the method described by Sarropoulou et al. [80]. The samples were incubated in a water bath at 80 °C until the plant material was fully discoloured (3–4 h). The absorbance of chlorophylls *a* and *b* was then measured at 665 and 649 nm, respectively. Total chlorophyll (Chl *a* + *b*) was determined according to Wintermans and de Mots [81] from the equations

Chl *a* + *b* [mg g^−1^ _FW_] = (6.10 ×A_665_ + 20.04 × A_649_) × V/1000/FW (1)

The absorbance of carotenoids (Cars) was measured at 440 nm. The carotenoids were estimated using the following equation:

Cars [mg g^−1^ _FW_] = (4.69 × A_440_ − 1.96 × A_665_ − 4.74 × A_649_) ×volume of supernatant (15 mL) × dilution factor/sample weight (0.1 g) (2)

The following three equations were used to calculate the content of protoporphyrin (Proto), Mg-protoporphyrin (Mg-Proto) and protochlorophyllide (Pchlide):

Proto [μg g^−1^ _FW_] = [(12.25 × A_665_ − 2.55 × A_649_) × volume of supernatant (mL)/sample weight (g)]/892 (3)

Mg-Proto [μg g^−1^ _FW_] = [(20.31 × A_649_ − 4.91 × A_665_) × volume of supernatant (mL)/sample weight (g)]/906 (4)

Pchlide [mg g^−1^ _FW_] = [(196.25 × A_575_ − 46.6 × A_590_ − 58.68 × A_628_) + (61.81 × A_590_ − 23.77 × A_575_ − 3.55 × A_628_) + (42.59 × A_628_ − 34.32 × A_575_ − 7.25 × A_590_)] × volume of supernatant (mL)/sample weight (g) × 1000 (5)

Carotenoid and porphyrin contents were estimated on a VIS spectrophotometer using the above equations as described by Lichtenthaler [67] and Porra et al. [82] and modified by Yang et al. [83].

### 4.9. ATR-FTIR Analysis

The study used attenuated total reflection Fourier-transform infrared spectroscopy (ATR-FTIR) to analyse the structural changes of dried plant samples. The plant material was dried in a laboratory dryer at 70 °C for 24 h. During the measurement, heat-dried leaves (test samples and control samples) were placed directly on the diamond crystal. The samples were scanned five times in the wave number range 600–4000 cm^−1^ using a PerkinElmer FT-IR/NIR Spectrometer Spectrum 3 with an ATR accessory with a temperature-controlled diamond window (MIR ATR Diamond). Each sample was scanned with 32 scans per sample and a resolution of 4 cm^−1^.

### 4.10. Statistical Analysis

For each parameter, data are presented as averaged values of at least four to eight biological replicates in each shoot and root variant. All data were analysed by Student’s *t*-test (differences between two variants—treated and not treated with bentonite—within a single binding agent) and one-way ANOVA followed by Tukey’s post hoc test (differences between three variants—treated with different binding agents—within a given treatment with bentonite). Differences were considered significant at *p* < 0.05. All statistical analyses were performed using Statistica 13.1 software (TIBCO Software, Palo Alto, CA, USA).

## 5. Conclusions

This study discussed the use of binders such as protein hydrolysates: fish collagen and keratin, and the biocide PHMB for the pre-sowing seed treatment of maize. The studies indicate that (i) elemental components such as Ca, Cu, Mg, Na, Mn and Zn accumulate to varying degrees in seeds, roots and shoots and may affect the metabolic processes of plant growth and development; (ii) bentonite, which was additionally applied, is a valuable nutrient source for plants due to its high content of macro- and microelements, thus supporting the growth and development of biomass; and (iii) the content of photosynthetic pigments and chlorophyll fluorescence parameters remained unchanged by the pre-sowing seed treatment. Our data suggest that the binders selected for seed treatment in the study may have a small effect on improving seed quality and maize seedlings growth and development. However, more research is needed in this area, including on the applied dose of biostimulant applied and testing different ratios of biostimulants used. Perhaps the results obtained, which do not clearly indicate the biostimulative nature of the seed coatings used, are the result of too short a period of experiments (seedling stage). However, it should be noted that the results obtained open up the possibility of further, more in-depth research on this subject, which concerns the search for new, environmentally friendly methods of increasing the yields of industrial crops in a way that is compatible with sustainable development.

## Figures and Tables

**Table 1:** Elemental composition of seeds, roots and shoots in 21-day-old maize plants. Values followed by different letters within the given binding agent treatment (NT, FC+P or KE+P) are significantly different (*p* < 0.05; ANOVA followed by Tukey’s post hoc test; *n* = 4)); values within a given bentonite treatment (H+B, FC+P+B or KE+P+B) are significantly different (* for *p* < 0.05, ** for *p* < 0.01, *** for *p* < 0.001; Student’s *t*-test; *n* = 4).

Variant	Ca [g kg^−1^ _DW_]	K [g kg^−1^ _DW_]	Mg [g kg^−1^ _DW_]	Na [g kg^−1^ _DW_]	P [g kg^−1^ _DW_]	Cr [g kg^−1^ _DW_]	Cu [g kg^−1^ _DW_]	Fe [g kg^−1^ _DW_]	Mn [g kg^−1^ _DW_]	Zn [g kg^−1^ _DW_]
**Seeds**										
NT	0.10 ± 0.0 a	9.0 ± 0.4 b	1.6 ± 0.0 ab	0.004 ± 0.0 a	4.8 ± 0.1 a	ND	0.0023 ± 0.0 a	0.04 ± 0.0 a	0.0181 ± 0.0 bc	0.0498 ± 0.0 a
H+B	0.25 ± 0.0 e ***	9.0 ± 0.4 b	2.0 ± 0.2 c *	0.37 ± 0.0 d ***	5.2 ± 0.1 a **	0.0013 ± 0.0 a	0.0027 ± 0.0 ab ***	0.06 ± 0.0 c***	0.0268 ± 0.0 d ***	0.0551 ± 0.1 ab
FC+P	0.16 ± 0.0 c	7.6 ± 0.0 a	1.6 ± 0.0 a	0.01 ± 0.0 c	4.9 ± 0.2 a	ND	0.0039 ± 0.0 c	0.06 ± 0.0 b	0.0135 ± 0.0 a	0.0635 ± 0.0 b
FC+P+B	0.24 ± 0.0 de ***	7.7 ± 0.1 a	1.8 ± 0.0 bc ***	0.57 ± 0.0 f ***	4.8 ± 0.3 a	0.002 ± 0.0 b	0.0029 ± 0.0 b ***	0.07 ± 0.0 c***	0.0293 ± 0.0 e ***	0.0541 ± 0.0 ab ***
KE+P	0.13 ± 0.0 b	7.4 ± 0.4 a	1.8 ± 0.0 bc	0.01 ± 0.0 b	4.9 ± 0.0 a	ND	0.0039 ± 0.0 c	0.06 ± 0.0 c	0.0157 ± 0.0 ab	0.0579 ± 0.0 ab
KE+P+B	0.24 ± 0.0 d ***	7.7 ± 0.1 a	1.8 ± 0.0 abc	0.40 ± 0.0 e ***	4.9 ± 0.2 a	0.0026 ± 0.0 c	0.0065 ± 0.0 d ***	0.14 ± 0.0 d***	0.0200 ± 0.0 c **	0.0511 ± 0.0 a **
**Roots**										
NT	23.3 ± 1.7 a	5.4 ± 0.0 b	1.6 ± 0.0 a	0.93 ± 0.0 a	1.3 ± 0.0 a	0.0049 ± 0.0 b	0.0710 ± 0.0 b	3.0 ± 0.0 a	0.0671 ± 0.0 a	0.0235 ± 0.0 a
H+B	32.6 ± 0.1 d ***	7.0 ± 0.0 d ***	1.9 ± 0.0 c ***	1.26 ± 0.0 a ***	1.8 ± 0.0 d ***	0.0051 ± 0.0 c ***	0.0079 ± 0.0 b ***	4.6 ± 0.0 f ***	0.0838 ± 0.0 f ***	0.0793 ± 0.0 e ***
FC+P	29.1 ± 0.3 b	6.0 ± 0.2 c	1.8 ± 0.1 b	2.79 ± 1.0 b	1.7 ± 0.0 c	0.0040 ± 0.0 a	0.0060 ± 0.0 a	3.5 ± 0.0 c	0.0694 ± 0.0 b	0.0280 ± 0.0 b
FC+P+B	31.7 ± 0.1 cd ***	4.1 ± 0.0 a ***	1.9 ± 0.0 bc	0.65 ± 0.0 a **	1.3 ± 0.0 a ***	0.0076 ± 0.0 d ***	0.0160 ± 0.0 c ***	3.4 ± 0.0 b **	0.0729 ± 0.0 c ***	0.0475 ± 0.0 c ***
KE+P	33.3 ± 0.1 d	5.8 ± 0.0 c	2.2 ± 0.0 d	0.75 ± 0.0 a	1.9 ± 0.0 e	0.0118 ± 0.0 f	0.0362 ± 0.0 d	3.6 ± 0.0 d	0.080.0 ± 0.0 d	0.0547 ±0.0 d
KE+P+B	30.9 ± 0.2 c ***	4.3 ± 0.0 a ***	1.8 ± 0.0 bc ***	0.47 ± 0.0 a **	1.3 ± 0.0 b ***	0.0082 ± 0.0 e ***	0.0401 ± 0.0 e ***	3.8 ± 0.0 e ***	0.0826 ± 0.0 e ***	0.0462 ± 0.0 c ***
**Shoots**										
NT	14.1 ± 0.1 c	56.6 ± 1.4 c	4.6 ± 0.1 bc	0.26 ± 0.0 b	12.1 ± 0.1 c	0.0029 ± 0.0 e	0.0101 ± 0.0 d	0.39 ± 0.0 d	0.0412 ± 0.0 c	0.1347 ± 0.0 c
H+B	14.5 ± 0.2 d *	50.2 ± 0.8 a ***	3.9 ± 0.0 a ***	0.43 ± 0.0 d ***	11.9 ± 0.2 c	0.0022 ± 0.0 c ***	0.0084 ± 0.0 c ***	0.37 ± 0.0 c ***	0.0375 ± 0.0 b ***	0.1586 ± 0.0 e ***
FC+P	14.0 ± 0.1 bc	53.0 ± 0.8 ab	4.4 ± 0.0 b	0.23 ± 0.0 a	11.6 ± 0.1 b	0.0013 ± 0.0 a	0.0066 ± 0.0 a	0.32 ± 0.0 a	0.0428 ± 0.0 d	0.1411 ± 0.0 d
FC+P+B	13.1 ± 0.1 a ***	55.1 ± 1.4 bc	4.8 ± 0.2 bcd *	0.31 ± 0.0 c ***	9.7 ± 0.0 a ***	0.0028 ± 0.0 d ***	0.0164 ± 0.0 f ***	0.33 ± 0.0 b**	0.0353 ± 0.0 a ***	0.1313 ± 0.0 b ***
KE+P	14.0 ± 0.0 c	52.7 ± 1.9 ab *	4.9 ± 0.3 cd	0.23 ± 0.0 a	11.4 ± 0.0 b	0.0056 ± 0.0 f	0.0150 ± 0.0 e	0.67 ± 0.0 e	0.0491 ± 0.0 e	0.1630 ± 0.0 f
KE+P+B	13.7 ± 0.1 b ***	51.6 ± 0.9 a	5.0 ± 0.1 d	0.27 ± 0.0 b ***	11.5 ± 0.1 b	0.0015 ± 0.0 b ***	0.0076 ± 0.0 b ***	0.39 ± 0.0 d ***	0.0429 ± 0.0 d ***	0.1258 ± 0.0 a ***

ND—not detected (below the detection thresh-old of used method). Macroelements: Ca, K, Mg, Na, P; microelements: Cr, Cu, Fe, Mn, Zn.

**Table 3:** Chemical composition of bentonite.

Element	Content (mg kg^−1^ _DW_)
Al	1669.7 ± 15.6
As	48.4 ± 0.9
B	233.0 ± 12.3
Ba	192.1 ± 7.9
Ca	3241.4 ± 214.2
Cr	7.8 ± 0.1
Cu	9.4 ± 0.1
Fe	5639.7 ± 214.2
K	1905.5 ± 49.1
Li	100.5 ± 0.8
Mg	940.5 ± 24.1
Mn	389.8 ± 4.6
Na	8804.8 ± 479.5
Ni	11.4 ± 0.1
Pb	17.9 ± 0.5
Sb	23.3 ± 0.8
Sr	64.8 ± 2.7
Ti	420.4 ± 3.7
V	2.4 ± 0.1
Zr	38.0 ± 0.7
Zn	45.9 ± 1.2

Ag, Bi, Cd, Co, Ge, Hg, Mo, Se, Sn—not detected (below the detection threshold of used method).

**Table 4:** Method of seed coating.

Acronym	Binder	Bentonite (B) Addition	Weight of 100 Seeds [g]	Amount of Binder or Binder and Bentonite Used to Envelope 100 Seeds [g]
NT	Non-treated	-	14.20 ± 0.08	-
H+B	H_2_O	+	14.05 ± 0.84	0.43
FC+P	Fish collagen + PHMB	-	14.98 ± 0.81	0.30
FC+P+B	Fish collagen + PHMB	+	14.78 ± 0.64	0.95
KE+P	Keratin + PHMB	-	15.52 ± 0.07	0.26
KE+P+B	Keratin + PHMB	+	14.47 ± 0.01	1.08

**Table 5:** Chemical composition of horticultural soil.

Available Nutrients	Content [mg kg^−1^ Soil]
P	392.4
K	538.1
Mg	1031.2
Mn	78.7
Cu	17.9
Zn	27.6
Fe	3296.9
Ca	17,411.7
Na	291.6
B	7.0

## Data Availability

The raw data supporting the conclusions of this article will be made available by the authors on request.
